# Valorization and food applications of okara (soybean residue): A concurrent review

**DOI:** 10.1002/fsn3.3363

**Published:** 2023-04-29

**Authors:** Aasma Asghar, Muhammad Afzaal, Farhan Saeed, Aftab Ahmed, Huda Ateeq, Yasir Abbas Shah, Fakhar Islam, Muzzamal Hussain, Noor Akram, Mohd Asif Shah

**Affiliations:** ^1^ Department of Home Economics Government College University Faisalabad Faisalabad Pakistan; ^2^ Food Safety and Biotechnology Laboratory, Department of Food Science Government College University Faisalabad Faisalabad Pakistan; ^3^ Department of Nutritional Sciences Government College University Faisalabad Faisalabad Pakistan; ^4^ Department of Economics Kebri Dehar University Jigjiga Ethiopia; ^5^ Division of Research and Development Lovely Professional University Phagwara India

**Keywords:** functional properties, nutritional profile, prebiotic, soybean residue, therapeutic potential

## Abstract

Agriculture waste is rising continuously across the globe due to enormous industrial, food processing, and household activities. Proper valorization of this waste could be a promising source of various essential bioactive and functional ingredients. Okara is a major residue produced as result of soybean processing and has a rich nutritional profile. The nutritional profile of okara is affected by the processing conditions, variety, pre‐treatment, post‐production treatments, and processing techniques. Owing to the high fibers, lipids, proteins, and bioactive components, it is being used as an essential industrial ingredient in various food processing industries. The prebiotic potential and nutritional profile can be increased by various techniques, that is, enzymatic, chemical, biotransformation, high‐pressure microfludization, and fermentation. The prebiotic potential of okara makes it suitable as a therapeutic agent to prevent a variety of metabolic disorders such as diabetes, obesity, hypercholesterolemia, and hyperlipidemia. The current review highlights the structural, nutritional, functional, therapeutic, and industrial applications of okara.

## Graphical abstract

This review emphasizes the prebiotic potential and nutritional profile of okara as well as different processes used to increase the nutritional profile of okara. The prebiotic potential of okara makes it a suitable therapeutic agent to prevent a variety of metabolic disorders such as diabetes, obesity, hypercholesterolemia, and hyperlipidemia. The current review highlights the structural, nutritional, functional, therapeutic, and industrial applications of okara.

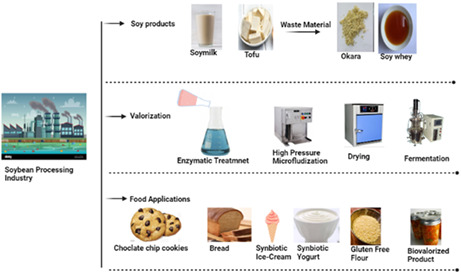

## 1 INTRODUCTION

Soybean is a member of *Leguminiaceae* (bean family) and is an important legume and oil crop. The consumption of soybean foods such as tofu, soymilk, and soy nuts has gained popularity in western and Asian regions. The processing of soy‐derived products has led to the production of soy residue; this soy residue is called okara. It is known as “honorable hull” or “soy pulp” in Japan, ‘douzha’ in China, and ‘biji’ in Korea (Li et al., 2012; Préstamo et al., 2007). Okara is an insoluble byproduct of soymilk, tofu, and soy‐nuts processing. Singapore, Hong Kong, Australia, Canada, the United States of America, South America, Europe, China, and India use soybeans and its processed products such as soymilk (Vong & Liu, 2016). Only a small amount of soy waste is used as animal feed, but a large portion is discarded as industrial waste. This was a large industrial waste and gained global attention in recent years because it has a good chemical composition, functional as well as anti‐nutrients such as saponins, tannins, phytic acids, and trypsin inhibitors (Li et al., 2012; Razavizadeh et al., 2021). Okara has a protein level of about 28%–30% (essential amino acid), a fat level of 8%–10% (polyunsaturated fatty acid), and a small number of other nutrients like starch, sugar, potassium, and some amount of B group vitamins that improve the digestion and make it a low‐cost plant‐based protein (Préstamo et al., 2007).

Recent reports showed the progress of okara from a residue to a functional or value‐added food. Biotransformation, which combines enzymatic saccharification and fermentation, is an efficient method of okara utilization. The biotransformed okara is more suited for additional processing to generate functional food items for human use, such as sauces or functional drinks, due to its higher levels of prebiotic dietary fiber, free sugars, and amino acids (Vong et al., 2017). The most common method to enhance the the sensory profile and functional qualities of okara is fermentation. Moreover, okara fermentation with probiotic bacteria improves the microbial viable count and storage of food products (Colletti et al., 2020). It contains aglycones and isoflavones as bioactive components. Fermentation of okara enhances its functional properties and may also improve its value because it contains lactose (as a prebiotic source) and probiotics (Voss et al., 2021). In this review, we suggest a possible use of okara as a healthy and functional food to improve gut function and prevent hyperlipidemia, hypertension, diabetes, cancer, and obesity.

### 1.1 Production technology

**FIGURE 1:**
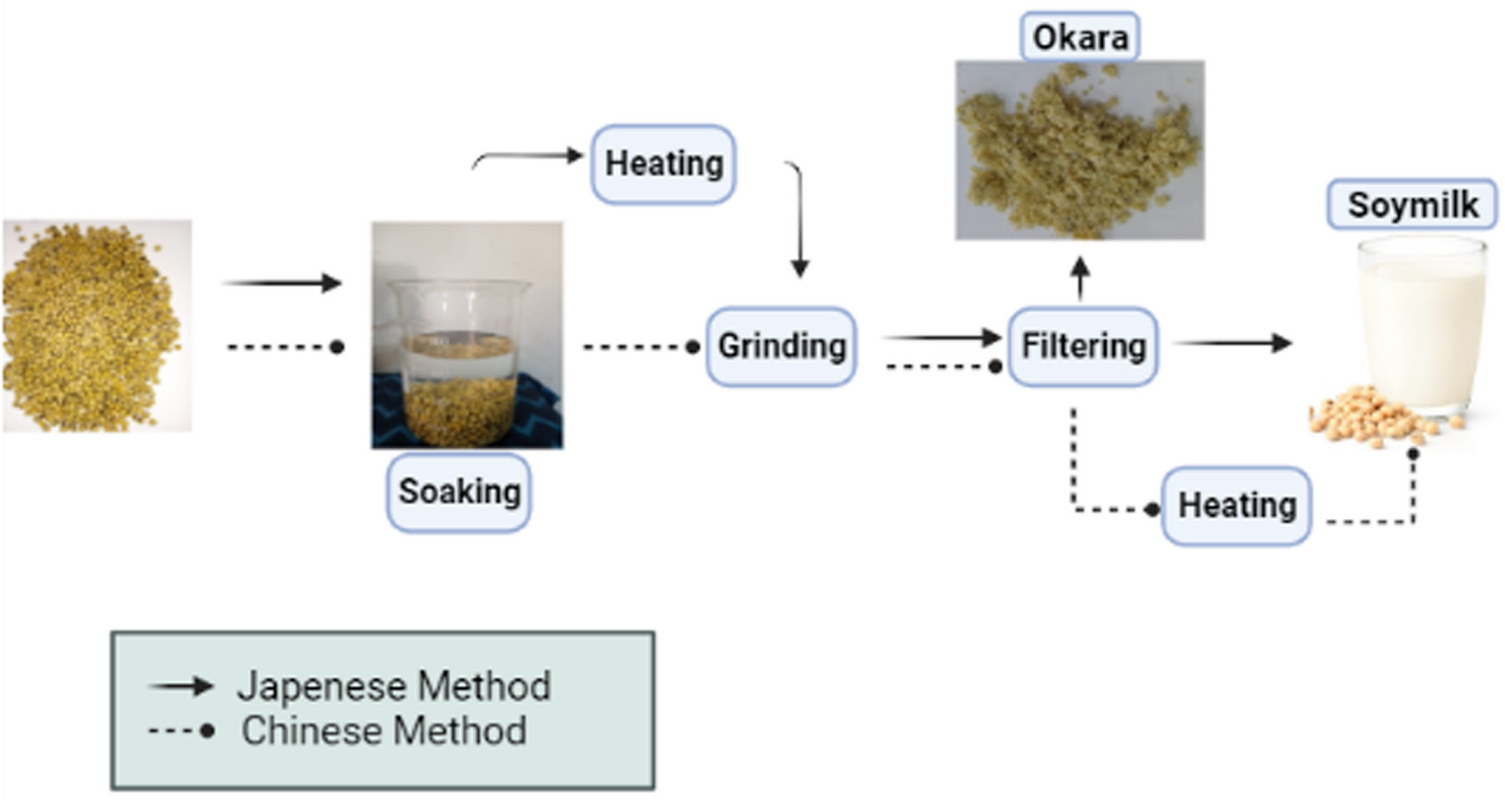
Extraction methods (Japanese and Chinese) of okara from soybean.

The composition of okara is determined by the quantity of water‐phase extraction from ground soybean, as well as the production of soybean. There are two methods commonly used for soymilk production as shown in Figure 1. The first is the Chinese method and the second is the Japanese method; there are very slight modifications in both methods. In the Chinese method, the following steps are followed during soymilk production: Soybean is first soaked, rinsed, and then ground and heated, and finally filtered. On the other hand, in the Japanese method, soybean is soaked, rinsed, and then heated, ground, and filtered to obtain soymilk (Figure 1). After filtration, the solid part that is leftover is known as okara (Guimarães et al., 2018). Okara is produced throughout the world. A 1 kg of tofu produced from soybean provides around 1.2 kg of fresh okara. Around 800,000 tons of okara is produced in Japan, 310,000 tons in Korea, and 2,800,000 tons in China by tofu industries. European nations too consume soybean in large quantities. Spain alone purchases 2.5 million tons of soybeans every year. These countries produce a large amount of soybean residue, okara (Li et al., 2012). A small portion of okara is recycled as animal feed. Okara‐based animal feed has good nutritional and bioactive components that improve the digestion of animals, the quality of meat, and milk production. It is found to be a cost‐effective and alternative source of protein and energy for animals (Bo et al., 2011; Rahman et al., 2021).

## 2 NUTRITIONAL COMPOSITION

Fresh okara is high in moisture (putrefies very fast) and dietary fiber content. Okara also is a rich source of protein and carbohydrates. Linoleic acid, palmitic acid, stearic acid, oleic acid, linoleic acid are the most common essential fatty acids present in okara. Okara contains monosaccharides, oligosaccharides, and polysaccharides such as arabinose, glucose, galactose, fructose, stachyose, raffinose, sucrose, and starch. Phytochemicals that are present in okara are phytates, saponins, coumestans, phytosterols, lignans, and isoflavones (genistein and daidzein). These chemicals have a variety of therapeutic and physiological properties, such as their antioxidant action, cardiovascular disease prevention, and are excellent chemo‐preventive agents for cancer patients (Li et al., 2012). Okara is also a rich source of minerals; every 100 g of okara contains 126 mg of Ca, 4.45 mg of Fe, 0.77 mg of Cu, 313 mg of phosphorus, 286 mg of potassium, and 3.14 mg of zinc (dos Santos et al., 2019; Kamble & Rani, 2020). Now a days, different varieties of soybean are grown (Black soybean, Yellow soybean); hence, the extracted okara has also different forms. The chemical compositions of black okara and yellow okara are shown in Table 1.

**TABLE 1:** Chemical composition of wet okara, dried okara, black soybean okara, and yellow soybean okara.

Chemical components	Wet okara	Dried okara	Black soybean okara	Yellow soybean okara	References
Moisture (%)	68.03	1	6.44	3.41	Anjum et al. (2022) Razavizadeh et al. (2021) Sengupta et al. (2012) Préstamo et al. (2007) Toda et al. (2007)
Fat (%)	6.02	12.6	16.46	7.21	
Protein (%)	8.08	34.15	34.38	31.67	
Carbohydrates (%)	12.01	48.9	38.53	24.42	
Dietary fiber (%)	5	33	7.51		
Ash (%)	1	2.05	4.83	4.46	
Total Solids (%)	32.05	95.04	–	–	

Okara is a rich source of dietary fiber. Dietary fiber is widely recognized to have a vital function in numerous physiological processes as well as in preventing health maladies (Li et al., 2012). There are four forms of fiber: crude fiber (CF), total dietary fiber (TDF), insoluble dietary fiber (IDF), and soluble dietary fiber (SDF) (Brownlee, 2011). Okara, like other vegetable wastes from the food sector, is high in IDF, but low in SDF. Different techniques such as enzymatic treatment, fermentation, micronization, and high‐pressure treatment improve the SDF concentration of okara (Han et al., 2008). Ultrasonication is a technique used to enhance the protein yield from okara. Extracted protein has a more zeta potential and yield (%); therefore, the protein isolate from okara is added in different food items (Eze et al., 2022).

### 2.1 Bioactive components

Some of the bioactive components of soybean are phenolics and isoflavones. Soy isoflavones are a part of flavones, an estrogen‐like plant chemical called phytoestrogen. These soy flavone compounds contain glycosides, for example, ferulic acid, genistein, chlorogenic, daidzein, and syringic acid. These phenolic components are important for physiological and health‐promoting functions. The amount of bioactive components in soybean depends upon the method for extraction and the temperature during processing. Okara contains 30% isoflavones, 28% glucosides, 15% aglycones, and 0.89% acetyl genistin.

Fermentation of okara converts the β‐glucosidases to aglycones which enhance the bioavailability of nutrients. Therefore, it can be used as value addition. Moreover, malonyl glucosides (19%), phytic acid (1.2%), saponins (0.10%), isoflavone glucosides (10.3%), acetyl glucosides (0.32%), and isoflavone aglycones (5.41%) are present in okara (Vong & Liu, 2016). Li et al. (2012) presented the bioactive profile of okara. They reported that aglycine 0.11 umol/g, glycitein 0.02 umol/g, genistein 0.04 umol/g, and daidzein 0.05 umol/g in dry form are present in okara. These are phytoestrogen compounds of the plant. These bioactive components of okara, especially isoflavones, prevent cardiovascular diseases, osteoporosis, cancer, and microbial infection, and enhance immunity and digestion. Isoflavones (genistein and daidzein), aglycones, and glycosides have strong antioxidant properties compared to other polyphenols. It is interesting to note that these antioxidants prevent DNA damage and low‐density lipoproteins oxidation. From the above discussion, it is clear that okara is a dominant source of bioactive components (Kamble & Rani, 2020).

## 3 STRUCTURAL CHARACTERISTICS

**FIGURE 2:**
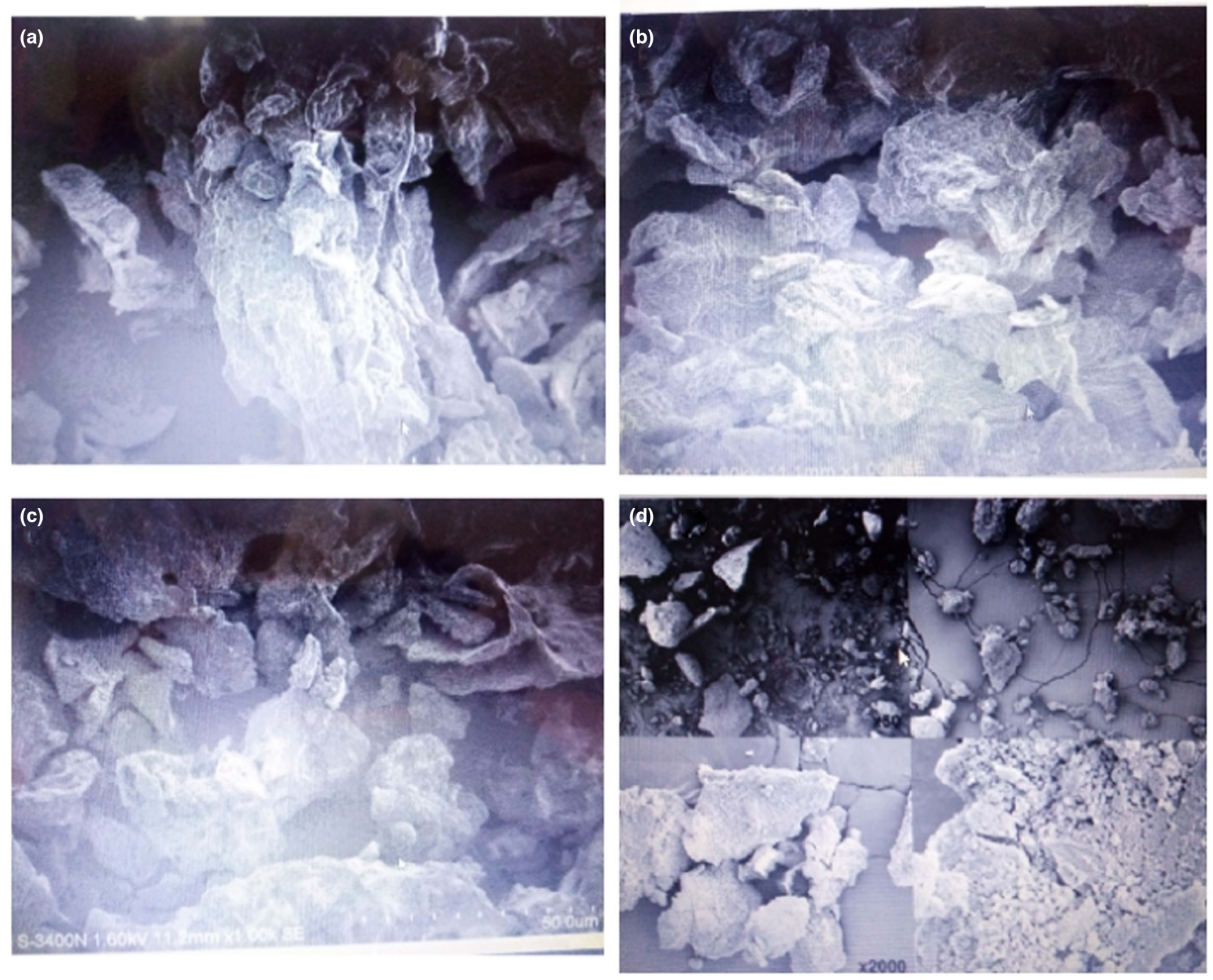
Scanning Electron Microscope images of okara dietary fiber (a), fermented dietary fiber (b), microwave‐treated dietary fiber (c), and okara flour (d). *Source*: Lin et al. (2020), dos Santos et al. (2019).

The structural properties of okara has been studied by various researcher using different techniques such as scanning electron microscope (SEM), Fourier transforms infrared spectroscopy (FT‐IR), X‐Ray diffraction (XRD), and energy dispersion X‐Ray analysis (EDX). Surface microstructure such as the arrangement of starch granules in the protein matrix and dietary components of okara was identified by SEM images as shown in Figure 2. The SEM images showed that okara powder has a rough, hollow, irregular, and porous structure and these describe the particle properties of the product. The rough and hollow structure represents okara containing IDFs (cellulose, hemicellulose); on the other hand, the irregular and porous structure of okara dietary fiber accounts for high water‐holding capacity and oil‐holding capacity. This describes okara as having hygroscopic properties. Dietary fiber extracted from okara mostly showed the crystalline and amorphous regions due to the presence of cellulose, hemicellulose, and lignins. Ostermann Porcel et al. (2017) studied the SEM images of bread enriched with different concentrations of okara. They determined that micrographs showed the relationship between bread and its composition. Due to the presence of protein and dietary fiber, okara‐enriched bread showed different micrographs.

The functional properties of okara may be identified by FT‐IR spectra, which showed structural similarities and differences in the product. The FTIR spectrum of frozen okara was studied by Quintana et al. (2017). They observed spectra at 2931, 2860, and 1750 cm^−1^, and these showed the presence of the CH_2_ group of hydrocarbons. This CH_2_ group represents okara and contains a carbonyl group of lipids and fatty acids. On the other hand, spectra at 1200 and 900 cm^−1^ represent the C–O–C bonds of glycosides. Glycosides show the presence of dietary fiber of okara. Another study by Lin et al. (2020) observed the FT‐IR spectra of okara dietary fiber. They reported different spectra of fermented dietary fiber, unfermented dietary and microwave‐treated dietary fiber of okara. The peak intensity and spectral lines of okara dietary fiber account for the functional groups of cellulose, hemicellulose, and lignins. The peaks near the 3401 cm^−1^ indicate the presence of O–H stretching vibration of the polymer and hydrogen bonding, which showed the existence of cellulose and hemicellulose. Due to the presence of C–H stretching, a peak near 2923 cm^−1^ was produced that indicates the cellulose polysaccharides. The 1637, 1061, and 877 cm^−1^ spectral lines are characteristic of glucuronic acid, C=O bonds, and β‐glycoside bonds, respectively. These bonds are present in cellulose and hemicellulose.

## 4 OKARA AS A SUBSTRATE

The functional, sensory, and microbial properties increased by the fermentation of okara (Table 2). The fermentation technique of plant media by certain lactobacillus species such as *Lactobacillus plantarum* and *Lactobacillus acidophilus* enhances the free radical scavenging activity and increases the nutritional value as well as the sensory, physical, and technological characteristics of okara (Razavizadeh et al., 2021). Fermentation had no discernible effect on the density of okara. After lactic acid fermentation, the oil‐holding capacity and water‐holding capacity levels significantly improved (Hu et al., 2019). The increase in oil‐holding capacity indicates that okara may be beneficial in food compositions where oil holding capacity (OHC) is decisive. Fermentation may be attributed to the physical binding of oil on the protein surfaces of okara. The increased hydrophobic amino acid availability on the protein surface induced by unrevealing and revealing of non‐polar amino‐acid residues from the core protein structures may be exploited to define the fermented okara (Çabuk et al., 2018). The breakdown of peptide bonds through fermentation owing to the proteolytic action might improve the number of polar groups and as a result, improve protein hydrophilicity (Akubor & Chukwu, 1999; Oloyede et al., 2016). Consequently, the greater water holding capacity (WHC) levels of fermented okara might be beneficial and related to enhanced solubility in water of okara proteins and increased water‐holding capacity of polar amino‐acid side‐chains (Razavizadeh et al., 2021). Guan et al. (2017) investigated whether solid‐state fermentation of okara with *Actinomucor Elegans* significantly increased the antioxidant, angiotensin‐converting enzyme (ACE) inhibition activity, and the release of peptide bonds by the degrading protein molecules of okara. They suggested that fermented okara can be used as a value‐added food. Various scientists reported the microbial properties of okara as shown in Table 2.

**TABLE 2:** Microbial characteristics of okara.

Treatment of okara	Study	Food product	Prebiotic effect	Health benefits	Reference
HHP (high hydrostatic pressure) + *Ultraflo*® L	In vitro study	Bakery Pastry industries Substitutes of cereal flours Gluten free flour for snacks	˄ SDF ˄ SCFA (short‐chain fatty acid) ˄ Lactic acid	˄ Growth of Good Bacteria ˄ Digestion	Pérez‐López et al. (2016)
Fermentation of okara	In vivo study	Supplemented food	˄ SDF ˄Oligosaccharide content ˄ short‐chain fatty acids	˅ Lipids ˅ Triglyceride ˄ Intestinal activity ˅ CVD (cardiovascular disease)	Villanueva‐Suárez et al. (2016)
HHP + *Ultraflo*® L enzyme	In vivo & in vitro study	High‐fat diet	˄ SCFA ˄ Dietary fiber	˄ Growth of Good Bacteria ˅ Weight ˄ Calcium and magnesium absorption	Pérez‐López et al. (2018)

*Note*: ˄ = increase, ˅ = decrease.

## 5 FUNCTIONAL CHARACTERISTICS

**FIGURE 3:**
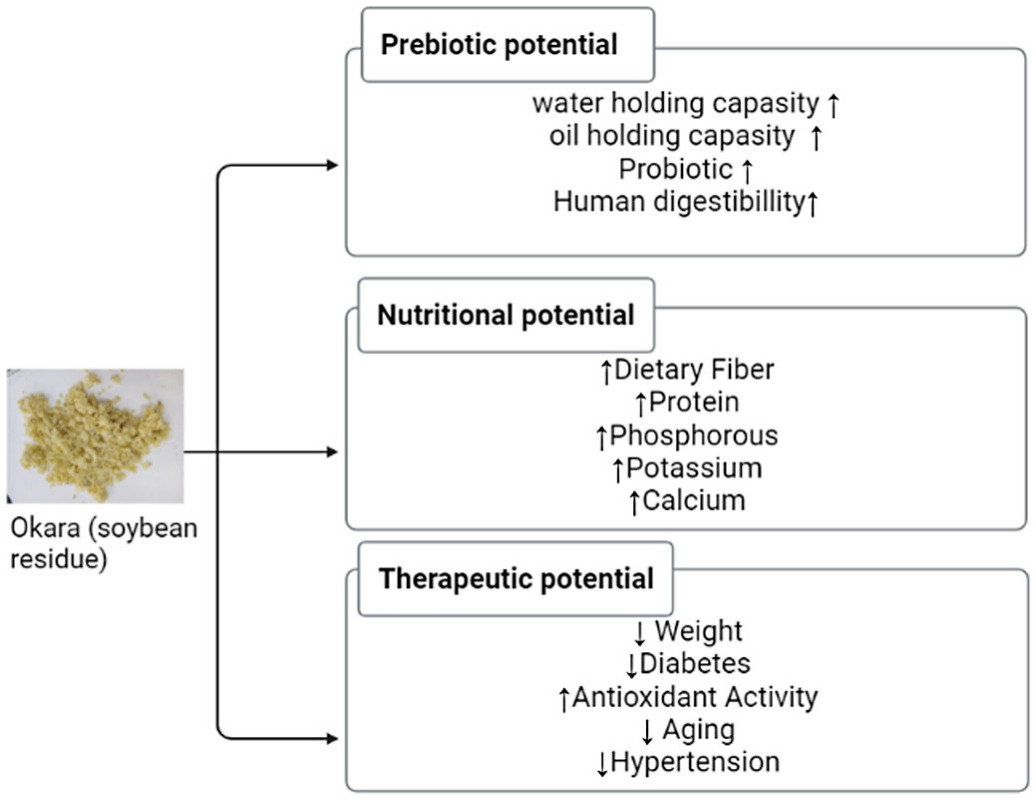
The prebiotic, nutritional, and therapeutic effect of okara.

Okara has isoflavone bioactive components which improve cancer resistance, protect from osteoporosis, reduce antimicrobial inflammation, and control cardiovascular disease. Intake of soy foods has been associated with a decrease in plasma cholesterol, the protection of heart disease, a lower risk of cancer (colon, breast, and prostate), osteoporosis, cognitive performance, and menopausal symptoms (Kamble & Rani, 2020). Fermented okara is used as a nutraceutical such as fucoxanthin and ecosapentaenoic acid (EPA) (Kim et al., 2023). Some prebiotic, nutritional and therapeutic effects of okara are shown in Figure 3.

### 5.1 Prebiotic potential

**FIGURE 4:**
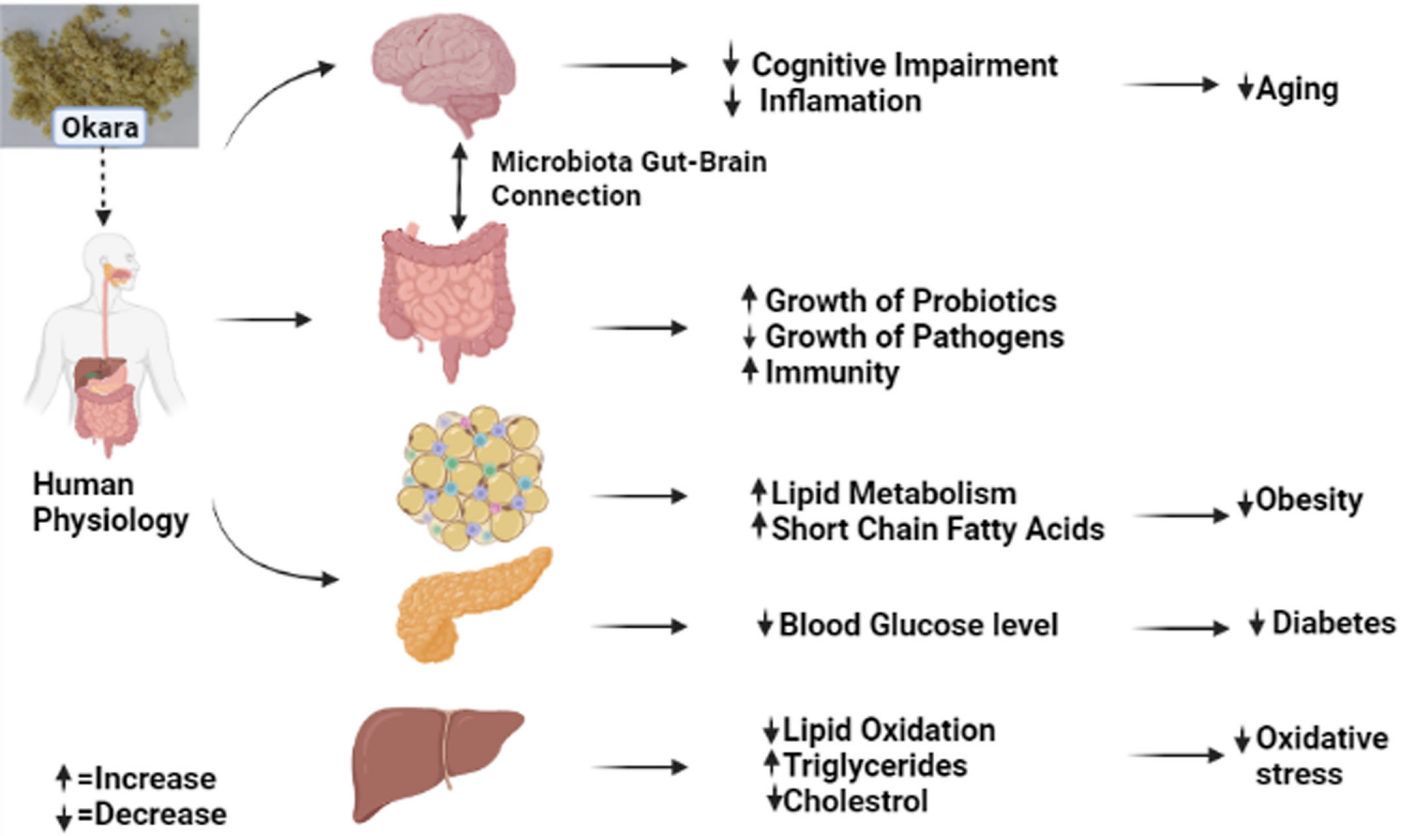
Effect of okara dietary fiber on human physiology (gut, pancreas, adipose tissue, and liver).

Prebiotics are nondigestable carbohydrates, and their breakdown starts within the small intestine while microbes in the large intestine absorb them. Endogenous and exogenous probiotics or commensal microorganism's growth is promoted by carbohydrates. Probiotics are live bacteria when consumed in sufficient quantities; it provides health benefits to the host. The increase of these microorganisms by prebiotics has several favorable consequences on host immunity and physiology. Therefore, dietary fiber, a source of prebiotics, is an important element of a healthy diet and has a crucial function in human physiology as well as in disease prevention (Corpuz et al., 2019). So, okara contains IDFs which are difficult to digest within the small intestine. Therefore, it is widely acknowledged as the primary substrate accessible for bacterial fermentation in the human colon. The human health benefits are associated with SDF and insoluble oligosaccharides like the management of metabolic problems associated with being overweight and preventing cancer. Soluble dietary fiber, galacto oligosaccharides, and inulin‐derived fructans (fructo‐oligosaccharides; FOS) are examples of common prebiotics. New prebiotics include xylo‐oligosaccharides, lactosucrose, polydextrose, malto‐oligosaccharides, gluco‐oligosaccharides, and soybean oligosaccharides (Pérez‐López et al., 2018). Okara is a plentiful and affordable byproduct of soybean that has the attractive perspective of prebiotics. Soybean and even okara have been applied with new technologies, such as high‐pressure microfluidization and fermentation of soybean waste by *Lactobacillus delbrueckii subsp*. and *bulgaricus* improve SDF by degrading insoluble polymers into simple carbohydrates (Pérez‐López et al., 2016). Figure 4 represents the effects of okara on human metabolism, especially in the small intestine, pancreas, adipose tissue, and liver.

### 5.2 Anti‐obesity

Okara is beneficial as a weight‐loss dietary supplement with a possible prebiotic impact, because okara is responsible for lowering body weight and more fecal fermentation (Préstamo et al., 2007). When Perez‐Lopez and his colleagues experimented and gave hypercholesterolemia diet with SDF‐enriched okara to rats, it resulted in various health‐promoting advantages. Due to the prebiotic potential of okara, it is needed for weight management and lipid reduction, increasing short‐chain fatty acid synthesis, improving mineral absorption, and modifying gut microflora. In comparison to untreated samples, in vivo colon digestion of okara results in lower pH, greater fecal weight, and increased short‐chain fatty acid (SCFA) synthesis in okara‐fed rats. For 10 weeks, mice that were fed an elevated fat diet (14% crude fat) or a dry okara‐added raised diet (10%, 20%, or 40%), it reduced the growth of body mass and endometrial white adipose tissue and avoided a rise in lipid levels, comprising cholesterol levels, relatively low cholesterol, and non‐esterified fatty acids (Matsumoto et al., 2007). Okara consumption also avoided hepatic steatosis. Okara consumption resulted in the upregulation of the cholesterol genes 7‐hydroxylase and downregulation of fatty acid synthesis genes in the liver, according to real‐time reverse transcriptase polymerase chain reaction. Figure 4 shows that okara helped to restore the normal microbiota to some extent and reversed the intestinal dysbiosis caused by high fat consumption reported by Pérez‐López et al. (2018).

### 5.3 Antidiabetic

Diabetes is categorized as a frequent metabolic disorder with complicated pathophysiology, described by persistent hyperglycemia. Diabetes is thought to be caused by several factors, such as impaired insulin production, impaired insulin physiological activity, and inflammatory response. Diabetic individuals' metabolisms may be altered, resulting in a variety of metabolic problems including sugar, protein, fat, water, and electrolytes (Chan et al., 2019). Furthermore, hyperglycemia may cause chronic damage and malfunction of different tissues including the eyes, kidneys, heart, blood vessels, liver, and nerves, resulting in catastrophic consequences. Therefore, diabetes is responsible for cell membrane damage, and changes in serum biochemical markers and lipid profile in diabetic patients. These changes can be reduced by the intake of an okara whey diet that reversed hyperglycemic conditions while also improving liver and pancreatic abnormalities. The chemical composition of soybean residue consists of 10 fat, 25% protein, 50% dietary fiber, and 15% other nutrients. Cytokines, lactoperoxidase, lactoferrin, lactoalbumin, and β‐lactoglobulin, as well as protein insulin, are called whey proteins and it includes different amino acids like valine, leucine, and isoleucine that are necessary for tissue repair and development. It also contains cysteine that has a free radical‐scavenging activity due to the presence of glutamic acid, glycine, and thiol groups all of which combine to make glutathione reductase (one of the cell's key antioxidants) (Kim et al., 2016; Usman et al., 2023). They also include vitamins, good protein fraction with strong water‐retaining and emulsifying properties, and an antihypertensive peptide. As a result, the combination of okara and whey protein has a therapeutic effect to reduce the hyperglycemic condition. So, okara can also be utilized as a nutritional supplement in biscuits and snacks to help reduce diabetes, hyperlipidemia, and obesity (Abdel‐Mobdy et al., 2021).

Nguyen et al. (2019) studied Vietnamese diabetes patients and they gave 6 g of okara dietary fiber for 2 weeks. They concluded that consumption of okara dietary fiber up to 60 g with different menus improves the blood glucose level. Therefore, different researchers claimed that okara dietary fibers have an excellent antidiabetic potential.

## 6 ANTIHYPERTENSIVE AND ANTIOXIDANT

Antioxidant amino acids such as phenylalanine, tyrosine, and methionine were found in higher quantities in all fermented okara products (Hwang et al., 2015). Biotransformation of okara improved its oxygen‐scavenging capacity by increasing the quantity of fiber in the diet, bioactive components (phenolic acids, isoflavones, aglycones), and amino acids (Vong et al., 2017). β‐carotene (0.411 mg/100 mL) and isoflavones (0.15 mol/gFM) were found in okara‐enriched food products which have antioxidant properties (Guimarães et al., 2018). Okara drinks (hydrolysates) inhibited ACE activity. Furthermore, antioxidant and ACE inhibitor action during in vitro intestinal digestion increased, whereas total phenolic components remained constant throughout all okara drinks (Sanjukta et al., 2015). Moreover, the current study has focused on the technique of boosting the antihypertensive action in milk or soy, but few studies have been undertaken using soy waste (Voss et al., 2021). Vital et al. (2018) showed that okara is an excellent source of phenolic acids, and flavonoids and are 106.7 mg GAE/100 g and 32.7 mg QE/100 g, respectively. They reported that the addition of okara to omega‐3 enriched milk can be used as an antioxidant supplement. Therefore, okara decreased lipid oxidation and increased the free‐radical scavenging activity.

### 6.1 Antiaging

Aging is a biological process defined as the organism's cellular and functional degradation with time and decreases the quality of life. Aging causes harmful modifications in tissues and cells with growing age, which increases the risk of diseases and death. Aging in accordance with this is the major hazard for the development of numerous ailments, including cardiovascular disease (e.g., stroke), cancer, and neurological disease (e.g., Alzheimer's disease) (Aman et al., 2021). The microbiota gut–brain connection is a bilateral communication channel that links the cognitive and emotional brain areas with peripheral intestinal functions. Microbiota's instability and increased endotoxin generation result in systemic inflammation and endotoxemia, which contribute to neuropsychiatric illnesses because gut flora plays a crucial role in metabolism and gastrointestinal inflammatory processes. Dysfunction of the gut microbes disturbed brain connection and it is related to the impairment of cognitive function and mood changes (Corpuz et al., 2019). Corpuz et al. (2019) have shown that taking probiotic strains and dietary bioactive components orally can reduce cognitive deficits in SAMP8 rats. The effect of okara supplementation on gut microorganisms was also studied to see if the extracted dietary fiber from okara can be eaten to produce an effect on the cognitive function by modifying the gut microbes or probiotics. When mice with accelerated aging eat okara, it assists to reduce the cognitive deterioration due to aging. Higher okara dietary fiber dosages may also play a substantial role in gut flora. Okara comprises isoflavones that have been proven to have neuroprotective and anti‐inflammatory properties. Moreover, genistein and 7,8,40‐trihydroxyisoflavone, a major constituent of daidzein, were demonstrated to improve cognitive function in rats via triggering the cholinergic pathway and the ERK/CREB/BDNF upregulating system. Okara enhanced cognitive function and revealed neuroprotective benefits in the hippocampus at lower okara dietary doses (7.5% okara) without changing gut flora. These findings suggest that okara can be a very effective daily neuroprotective food for the reduction of aging and cognitive impairment (Corpuz et al., 2019; Wang et al., 2020).

### 6.2 Anti‐hyperlipidemia

Okara has potential for the management of hyperlipidemia. A male golden Syrian hamster was fed a high‐fat supplemented diet with okara for three weeks (Kitawaki et al., 2009). The diets comprised either 13% or 20% of okara fiber (OK‐13 and OK‐20), low‐protein okara containing 13% fiber (OK1‐13), and isoflavones‐free okara with 13% fiber (OK2‐13). In hamsters which were given OK‐20, plasma concentration of triglycerides, extremely low‐density lipids, as well as cholesterol and low‐density lipid cholesterol levels dropped considerably (*p* < .05). In all tests, however, no substantial differences between low‐density lipid cholesterol and high‐density lipid cholesterol plasma (*p* > .05) concentrations were identified (Villanueva et al., 2011). The OK‐20 diet decreased total triglycerides, fats, and esterified cholesterol levels within the liver. All the okara foods tested the improved fecal output of total circulating free cholesterol, triglycerides, lipids, and overall nitrogen (*p* > .05). The findings revealed that the primary contents of okara such as fiber and protein might be responsible for the reduction of cholesterol and lipids within plasma and liver, and an enhancement in fecal excretion in high‐fat supplemented hamsters.

### 6.3 Food applications of okara

Okara has been utilized as a dish or supper in China and Japan for numerous years. It is quite simple to add fiber and protein to food to help meet nutritional content claims. Okara can bind moisture and oil, making it an appealing low‐price additive for increasing meat product production. The addition of okara (5%) in chocolate chip cookies increased its shelf life and decreased the syneresis during freezing and thawing. Okara fortified and supplement meat and bread can be used because it cannot change the flavor and texture of food (Mateos‐Aparicio et al., 2010). Ibrahim et al. (2022) reported that 2% and 3% okara fortification with probiotics (L. plantarum) and ice‐cream significantly improved its chemical, nutritional, microbial, physical, and sensory properties. They called this food product synbiotic ice‐cream because it enhances the growth of probiotics as well as nutritional composition. At the same time, Roslan et al. (2021) fortified yogurt with a probiotic (*L. Plantarum*) and okara (1%, 2%, and 3%). They also concluded that okara dietary fiber improves the probiotic count and chemical properties of yogurt with storage time. Therefore, okara can be used in food industries as a value‐added food and as a supplement food.

Furthermore, freeze‐dried okara has the highest swelling, lipid‐binding, and water‐holding abilities, usually accompanied by hot air drying and vacuum drying. Hot air‐dried okara has the highest cation exchangeability, as compared to freeze‐drying and vacuum‐drying okara (Li et al., 2011). Okara can be used in soy flour, wheat flour, and other components in food manufacturing to boost fiber and protein content. It has been used in the production of bread, pancakes, puffed noodle, food, confectionery, sausage, beverage, and nutritive flour. Production of bread with okara flour and wheat flour showed good nutritional and sensory properties. The crust color of bread changed significantly and improved the protein, fiber, and caloric contents (15 Kj/g). This bread could be used as a substitute food for diabetic patients (Wickramarathna & Arampath, 2003). Rotem and Almog (2009) produced a protein‐enriched premix powder incorporating okara for use in healthy foods. Dried milled okara (70 mesh) was combined with gluten in several proportions ranging from 3/1 to 12/1. The product comprised soy protein ranging from 10% to 30% and protein content ranging from 15% to 50%. Ostermann Porcel et al. (2017) described the gluten‐free properties of okara flour or dried okara. Okara flour contains high contents of fiber, and protein. Microwave treatment can alter the functional properties of okara. Asghar et al. (2022) studied the chemical properties of okara and then added 3% okara with probiotics in yogurt. In this experiment, okara used as a prebiotic and yogurt has antioxidant as well as probiotic property. They formed synbiotic yogurt by adding 3% okara with probiotic (*lactobacillus Rhamnosus*). The above studies clearly show that the addition of okara in any food item can increase the shelf life of the product as well as the nutritional and functional characteristics of the food product.

## 7 CONCLUSION

Even though okara is a byproduct of soybean, it includes numerous other useful components. According to the findings of many studies, okara can be utilized as a functional food. It has a high‐quality nutritional profile, phytochemicals, and prebiotics potential. In this article, okara has been assessed as a food ingredient enriched with prebiotics, high fiber, protein, fat, digestible carbohydrates, moisture content, and bioactive components, as well as food industry application. Benefits of okara include weight loss, blood glucose management, cognitive performance, ACE inhibition, free radical scavenging activity, and lipid reduction as well as a prebiotic‐like effect due to its capacity to increase SCFA synthesis, improve mineral absorption and modify gut flora. Okara can be used as a value‐added food, as a supplement, and as a fortified food.

## AUTHOR CONTRIBUTIONS

**Aasma Asghar:** Methodology (equal); writing – original draft (equal). **Muhammad Afzaal:** Conceptualization (equal); supervision (equal). **Farhan Saeed:** Conceptualization (equal); writing – review and editing (equal). **Aftab Ahmed:** Conceptualization (supporting); methodology (equal). **Huda Ateeq:** Data curation (equal); writing – review and editing (equal). **Yasir Abbas Shah:** Writing – review and editing (equal). **Fakhar Islam:** Formal analysis (equal); software (supporting). **Muzzamal Hussain:** Software (equal). **Noor Akram:** Validation (equal); visualization (equal). **Mohd Asif Shah:** Software (equal); writing – review and editing (equal).

## FUNDING INFORMATION

The authors declare that no funds, grants, or other support were received during the preparation of this manuscript.

## CONFLICT OF INTEREST STATEMENT

The authors declare that they have no conflict of interest.

## ETHICS STATEMENT

This article does not contain any studies with human participants or animals performed by any of the authors.

## CONSENT TO PARTICIPATE

The corresponding author and all the co‐authors participated in the preparation of this manuscript.

## INFORMED CONSENT

For this type of study, formal consent is not required.

## Data Availability

Even though adequate data have been given in the form of tables and figures, all authors declare that if more data are required, then the data will be provided on a request basis.
